# The Treatment of Wounds, Ulcers, and Abscesses

**Published:** 1895-03

**Authors:** 


*The Treatment of Wounds, Ulcers, and Abscesses.* By W. WATSON CHEYNE, M.B., F.R.S. Pp. xii., 197. Edinburgh: YOUNG J. PENTLAND. 1894.

In this small book the author does not attempt to discuss the whole subject of the treatment of wounds, or the various plans which are used by different surgeons, but contents himself with describing in simple language the method which he himself employs, and has found to be the simplest consistent with certainty in the results. The text of the book may be found in a sentence placed at the beginning, "Suppuration occurring in a wound made by a surgeon through unbroken skin is due to some oversight on his part." Dr. Watson Cheyne is a well-known advocate of the Listerian or antiseptic method of treating wounds, rather than that adopted by many Continental surgeons, notably in Germany, of attempting rigid asepsis as opposed to the employment of antiseptics. He points out the extreme difficulty of carrying out a rigid asepsis, especially in hospital practice, where it has even been found necessary to separate the auditorium from the area of an operation theatre by a wall of plate-glass! In well-marked contrast with such elaborate details as are here involved, the plan recommended by the author is as simple as possible. He does not even consider it necessary to boil instruments or suture-materials, but considers that immersion for two or three hours in a 5 per cent. solution of carbolic acid is quite sufficient. He does not agree with Mr. Lockwood that the ordinary methods of disinfecting the skin are quite inefficient, but says: "It is quite evident that some error in the bacteriological methods has crept into Mr. Lockwood's experiments." The book throughout bears evidence of the truly scientific spirit. It is written by one who has worked largely at bacteriology as well as practical surgery, and we quite agree with him that a knowledge of the former is absolutely necessary at the present day for the successful working of the latter.

